# Localized bursting of mesocarp cells triggers catastrophic fruit cracking

**DOI:** 10.1038/s41438-019-0161-3

**Published:** 2019-06-22

**Authors:** Eckhard Grimm, Jan Hahn, Daniel Pflugfelder, Moritz Jonathan Schmidt, Dagmar van Dusschoten, Moritz Knoche

**Affiliations:** 1 Abteilung Obstbau, Institut für Gartenbauliche Produktionssysteme, Leibniz Universität Hannover, Herrenhäuser Straße 2, 30419 Hannover, Germany; 2 Laser Zentrum Hannover e.V., Hollerithallee 8, 30419 Hannover, Germany; 3 IBG-2: Pflanzenwissenschaften, Forschungszentrum Jülich, Wilhelm-Johnen-Straße, 52428 Jülich, Germany

**Keywords:** Plant sciences, Plant physiology

## Abstract

The so-called rain-cracking of sweet cherry fruit severely threatens commercial production. Simple observation tells us that cuticular microcracking (invisible) always precedes skin macrocracking (visible). The objective here was to investigate how a macrocrack develops. Incubating detached sweet cherry fruit in deionized water induces microcracking. Incubating fruit in D_2_O and concurrent magnetic resonance imaging demonstrates that water penetration occurs only (principally) through the microcracks, with nondetectable amounts penetrating the intact cuticle. Optical coherence tomography of detached, whole fruit incubated in deionized water, allowed generation of virtual cross-sections through the zone of a developing macrocrack. Outer mesocarp cell volume increased before macrocracks developed but increased at a markedly higher rate thereafter. Little change in mesocarp cell volume occurred in a control zone distant from the crack. As water incubation continued, the cell volume in the crack zone decreased, indicating leaking/bursting of individual mesocarp cells. As incubation continued still longer, the crack propagated between cells both to form a long, deep macrocrack. Outer mesocarp cell turgor did not differ significantly before and after incubation between fruit with or without macrocracks; nor between cells within the crack zone and those in a control zone distant from the macrocrack. The cumulative frequency distribution of the log-transformed turgor pressure of a population of outer mesocarp cells reveals all cell turgor data followed a normal distribution. The results demonstrate that microcracks develop into macrocracks following the volume increase of a few outer mesocarp cells and is soon accompanied by cell bursting.

## Introduction

Rain cracking of fleshy fruit imposes a severe limitation on production and is associated with enormous commercial losses worldwide. Sweet cherries and grapes are the most notable examples of this problem but numerous other fleshy fruits are susceptible to rain cracking including, blueberry, currant, plum, sour cherry and tomato (for review, see ref. ^1^). The coincidence of rainfall and fruit cracking suggests a causal relationship and, hence, that such cracking is associated with a change in fruit water relations linked to surface wetness.

At this stage, two alternative hypotheses seek to explain the relationship between fruit cracking and rainfall^2^. The traditional “balloon” hypothesis (also referred to as the “critical turgor” hypothesis) assumes a fruit to resemble a thin-walled pressure vessel (the taut skin) containing a semi-fluid matrix (the flesh) which is approximated by a solution having a very negative osmotic potential. Water inflow may occur through the skin during periods of surface wetness (rain or dew) and/or through the vasculature of the fruit stalk. This water inflow increases the pressure in the fruit flesh, the volume of the fruit and, hence, the fruit’s surface area. The tangential skin strain causes tangential skin stress. The fruit cracks when the limits of skin strain (extensibility) and the critical skin stress (tension) are exceeded^3^. While this hypothesis offers a plausible, easy to understand and straightforward explanation of fruit cracking, more recent experimental evidence has become available that suggests a more complex mechanism is involved. This evidence includes (1) the lack of significant turgor in a cracking-susceptible grape^4–6^ and in sweet cherry^7,8^, (2) the initiation of cracking in the absence of a net uptake of water^9,10^, (3) vastly different cracking percentages for fruit that are forced to take up water through the skin as compared to fruit taking up the same amount of water via a perfusion method^10^, (4) the lack of any turgor increase with water uptake at the growth stages when cracking occurs^7^ and (5) the skin’s low modulus of elasticity, indicative of a skin that expands (strains) relatively easily.

The so-called “zipper” hypothesis for fruit cracking was proposed fairly recently. It offers an alternative explanation for fruit cracking^11^. According to this more recent hypothesis, cracking results from very localized phenomena, i.e. a local skin defect. Here, a local defect causes a zipper-type propagation of a microcrack to form a macroscopic crack—just as a “ladder” runs in a fine, knitted fabric^12^. A cuticular microcrack is likely to represent the initial local skin defect^13^. Microcracks are limited to the cuticle and—in general—do not extend into the underlying epidermis, hypodermis and mesocarp parenchyma. They are barely detectable by naked eye, but seen with the aid of a microscope ideally following infiltration with the fluorescent tracer acridine orange^2,13^. Such a microcrack impairs the local barrier properties of the cuticle, allowing rapid, localized, water uptake^9^. This results in the bursting of an individual epidermal cell in the near vicinity of the microcrack. The mechanical failure of an individual epidermal cell results in a concentration of stresses in the taut skin which focuses on the immediately adjacent epidermal cells. Moreover, the burst epidermal cell releases its contents into the cell-wall free space (apoplast) exposing the cell-wall material to high concentrations of malic acid (a major constituent of the sweet cherry symplast). The malic acid weakens the cell wall by decreasing its pH and by releasing cell-wall-bound Ca. The result is a swelling of the cell wall^12,14^. Because malic acid also increases cell membrane permeability, the initially very local defect “runs” (propagates) and the skin progressively “unzips” eventually leading to a fruit macrocrack (a gross crack, plainly visible to the naked eye). This hypothesis is consistent with: (1) the lack of high fruit turgor, (2) the cracking of fruit following localized exposure to surface water, despite a net loss of fruit water, and (3) the occurrence of tensional strain in the skin resulting from growth stress.

The evidence for the bursting of individual epidermal cells is indirect. It is based on the observations that: (1) epidermal cells have less negative osmotic potentials and, hence, water potentials than flesh cells^15^, (2) cell walls swell upon plasmolysis^15^ and (3) cells with swollen walls are easily separated due to decreased cell:cell adhesion. This is indexed by a decrease in fracture tension and a changed failure mode, with separation along cell walls, rather than across them^12^. In addition, anthocyanins are reported to leak into the bathing solution when a sweet cherry fruit is incubated in water. Anthocyanins being normally resident in the vacuole, this indicates bursting of whole flesh cells^16^. To obtain further evidence in support of the zipper hypothesis requires a finer spatial resolution of water flows within the fruit and the measurement of dimensional changes of individual cells before and during fruit cracking.

The objectives of the present study are: (1) to study the development of a macrocrack that is characteristic of fruit cracking, (2) to monitor water inflow through a microcrack into the underlying tissues and (3) to quantify the dimensional changes of cells of the outer mesocarp in the vicinity of a developing macrocrack and (for comparison) at some distant from this crack.

We used magnetic resonance imaging (MRI) to follow uptake of heavy water (D_2_O) into a submerged sweet cherry fruit and optical coherence tomography (OCT) to quantify the dimensional changes in the tissue with cell-level resolution. Microcracks detected by OCT begin to extend into epidermal and hypodermal cell layers. They develop into macroscopic cracks when progressing tangentially and radially into the flesh. Sweet cherry was used as a model, because of the amount of detailed information already available to us on its fruit-skin morphology, mechanics and water relations.

## Results

**Fig. 1:**
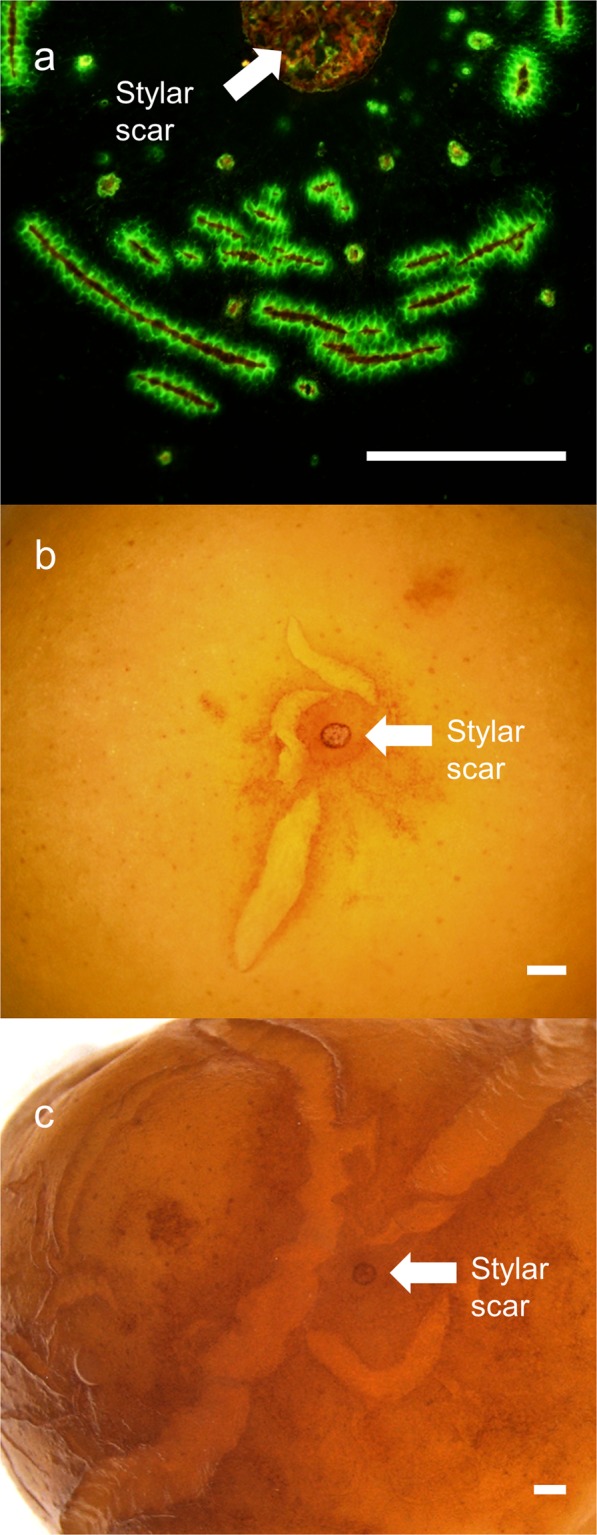
From microcracking to macrocracking of sweet cherry fruit. **a** Microcracks in the stylar scar region as indexed by acridine orange infiltration and fluorescence microscopy. **b**, **c** Stylar scar region after incubation periods of 9 h (**b**) and 24 h (**c**) in deionized water. Microcracks had extended into macroscopically visible cracks. Bars = 1 mm

Incubating fruit in deionized water induced microcracking. These microcracks allowed penetration of the fluorescence tracer acridine orange into the underlying tissue as indexed by the formation of a greenish halo along the microcracks (Fig. 1a). Microcracks in the stylar scar region were always oriented normally to the longitudinal axis of the fruit. As incubation continued, the frequency of microcracks in the stylar scar region increased. Later on, microcracks developed into macrocracks that propagated into the cheek region (Fig. 1b, c).

**Fig. 2:**
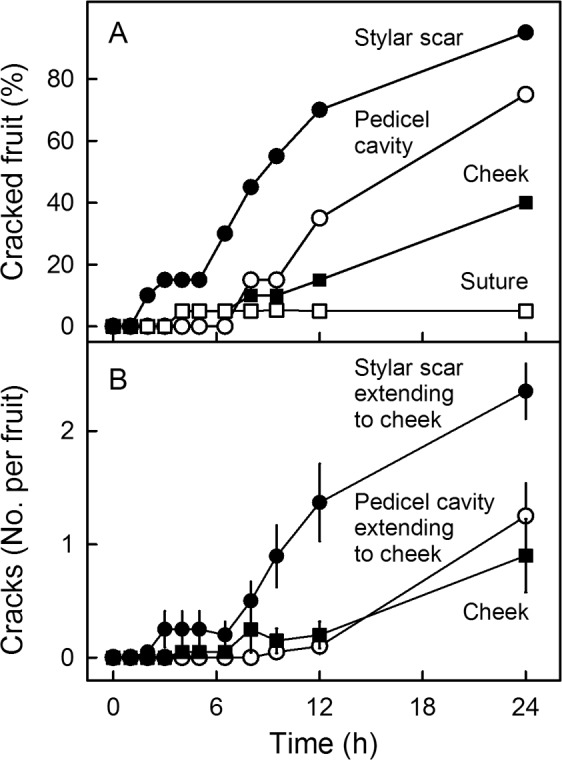
Time course of cracking of sweet cherry fruit. **a** Frequency of macrocracks in different regions of the fruit surface. **b** Origin of macrocracks on the cheek. Most macrocracks on the cheek resulted from extension of cracks from stylar scar and pedicel cavity regions. Data represent means (**a**) or means ± standard errors (**b**)

The frequency of macrocracks was highest in the stylar scar region, followed by pedicel cavity and cheek region (Fig. 2a). Most macrocracks in the cheek region propagated from ones initiated in the pedicel cavity or stylar scar regions (Fig. 2b).

**Fig. 3:**
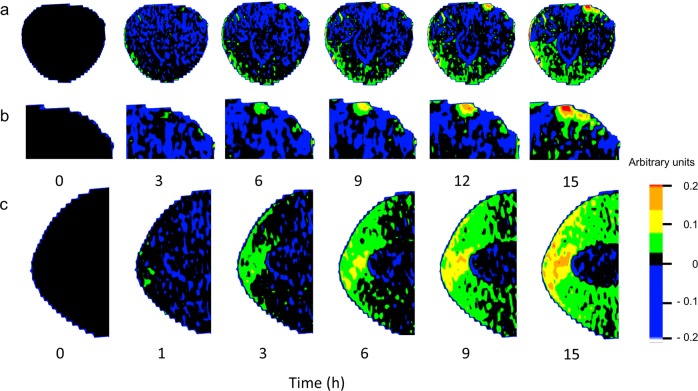
Time courses of crack formation and D_2_O uptake into sweet cherry fruit as quantified by magnetic resonance imaging (MRI). **a** Longitudinal cross-section along the pedicel/stylar scar axis. A microcrack had formed within 1 h of incubation in the pedicel cavity and the stylar scar regions. **b** Same as (**a**) but a view of the pedicel cavity at higher magnification. **c** Same as (**a**) but a view of the stylar scar region. Images reveal progressive influxes of water as the color changes from black/blue to green to yellow and orange

Within 1 h of incubation, water (D_2_O) penetration occurred through microcracks that had formed in the pedicel cavity and the stylar scar region (Fig. 3). The remaining fruit skin was without detectable label. The amount of water penetration in the stylar scar region was considerably greater than that in the pedicel-cavity region. Furthermore, the entire stylar scar region was affected by water penetration, whereas in the pedicel-cavity region the effects remained localized (Fig. 3b, c).

**Fig. 4:**
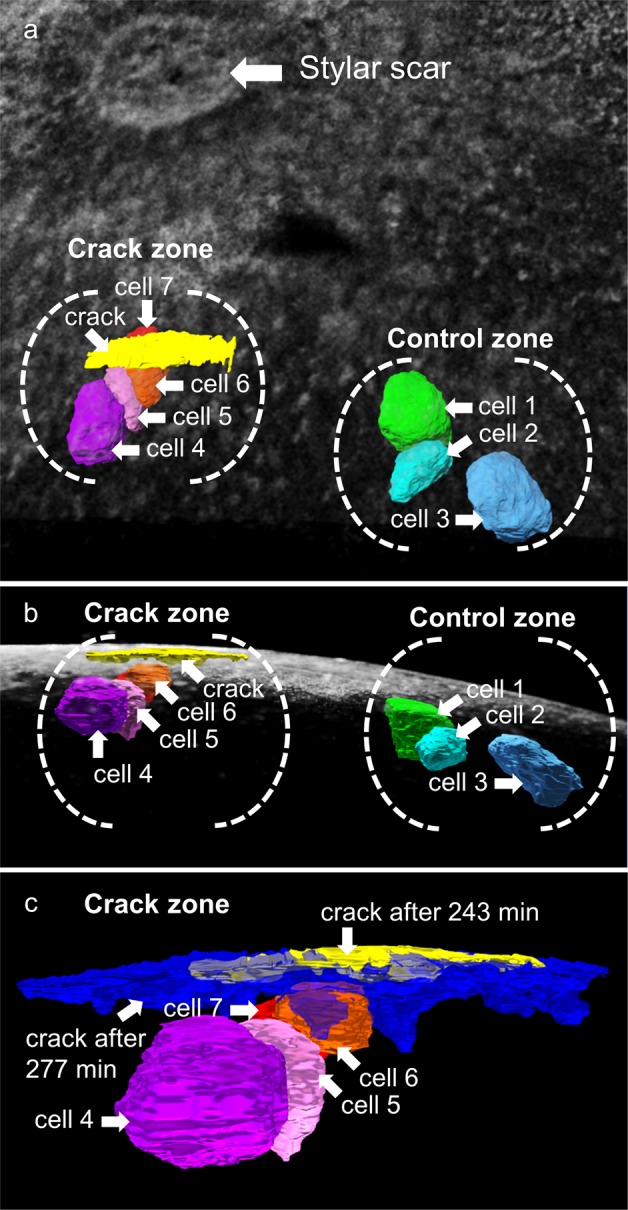
Stylar scar region of sweet cherry fruit as viewed by optical coherent tomography (OCT). **a**, **b** A microcrack that developed into a macrocrack (yellow color) had formed beneath the stylar scar. The image was taken after 243 min of incubation in deionized water. Position of outer mesocarp cells underneath the crack (crack zone) and in some distance from the crack (control zone). **a** Top view. **b** Cross-section. **c** Same view as (**b**) but limited to the crack zone after 277 min of incubation demonstrating the extension of the crack (blue color) relative to the initial crack (yellow color). The crack progressed in tangential and radial directions between mesocarp cells along the cell walls

The data from the OCT scan allowed computation of three-dimensional images of the crack zone of whole sweet cherry fruits (Fig. 4). A microcrack that later developed into a macrocrack was detected near the stylar scar by OCT after about 200 min of incubation in water (Fig. 4a). Two groups of cells in the outer mesocarp were selected to assess cell dimensional change before and after cracking. Cells 1, 2 and 3 were located in the control zone at some distance from the developing macrocrack, cells 4, 5, 6 and 7 in the crack zone. Changes in the volume of the crack and of the cells (1 through 7) were followed over time.

**Fig. 5:**
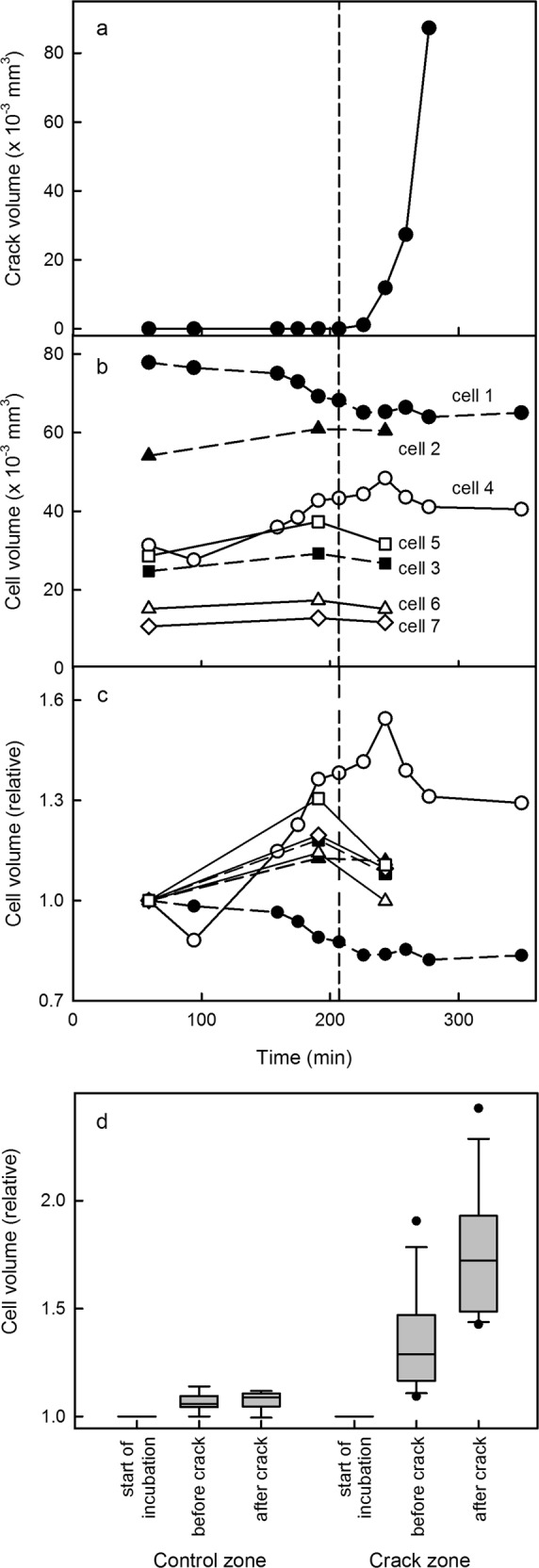
Time course of changes in cell volumes during cracking. **a−c** Time courses of change in volume of a microcrack that develops into a macrocrack (**a**) and in absolute volume (**b**) and relative volume (**c**) of cells of the outer mesocarp in the crack zone beneath a developing macrocrack and in the control zone at some distance from the crack. The exact location of the cell relative to the crack is identified by the number of the cell in Fig. 4. **d** Relative change in volume of cells in the control zone and in the crack zone during the initiation period (“start of incubation”), a maximum of 30 min before (“before crack”) and after the microcrack developed into a macrocrack (“after crack”). The box plot in (**d**) represents 9 and 12 observations in the control and the crack zone, respectively. The relative change in volume of cells was calculated by dividing the cell volume at time *t* by the volume of the same cell at the onset of the incubation period (*t* = 0–30 min). Data symbols in (**a−c**) represent individual cracks (**a**) or cells (**b**, **c**)

In subsequent scans obtained about 30 min later, the developing crack extended tangentially and radially towards the pit as indicated by the blue color relative to its initial length in yellow. The microcrack (yellow) had developed into a macrocrack (blue) (Fig. 4). Quantifying the volume of the crack indicated an exponential increase in volume following its first detection as a microcrack after about 200 min of incubation (Fig. 5a). During the pre-crack phase (i.e. from 0 to 200 min) the volumes of most cells increased gradually; the exception was cell 6. The increases in volume were not uniform, being largest for cells 4 and 5 (Fig. 5b, c). These two cells were located just beneath the site where the microcrack was observed a few minutes later (Fig. 4b, c). There were only small increases in volume of the cells in the control zone despite the significant opening and extension of the microcrack to a developing macrocrack. Statistical analyses of several crack events revealed significant differences in the changes in cell volume between control zone and crack zone (Fig. 5d). Within the control zone, cell volume increased slightly during incubation in water. There was essentially no significant difference in cell volume before and after formation of a microcrack. In contrast, cells in the crack zone revealed a 1.3-fold increase in cell volume before and a further 1.7-fold increase in cell volume after the microcrack was detected and extended into a macrocrack, respectively.

**Fig. 6:**
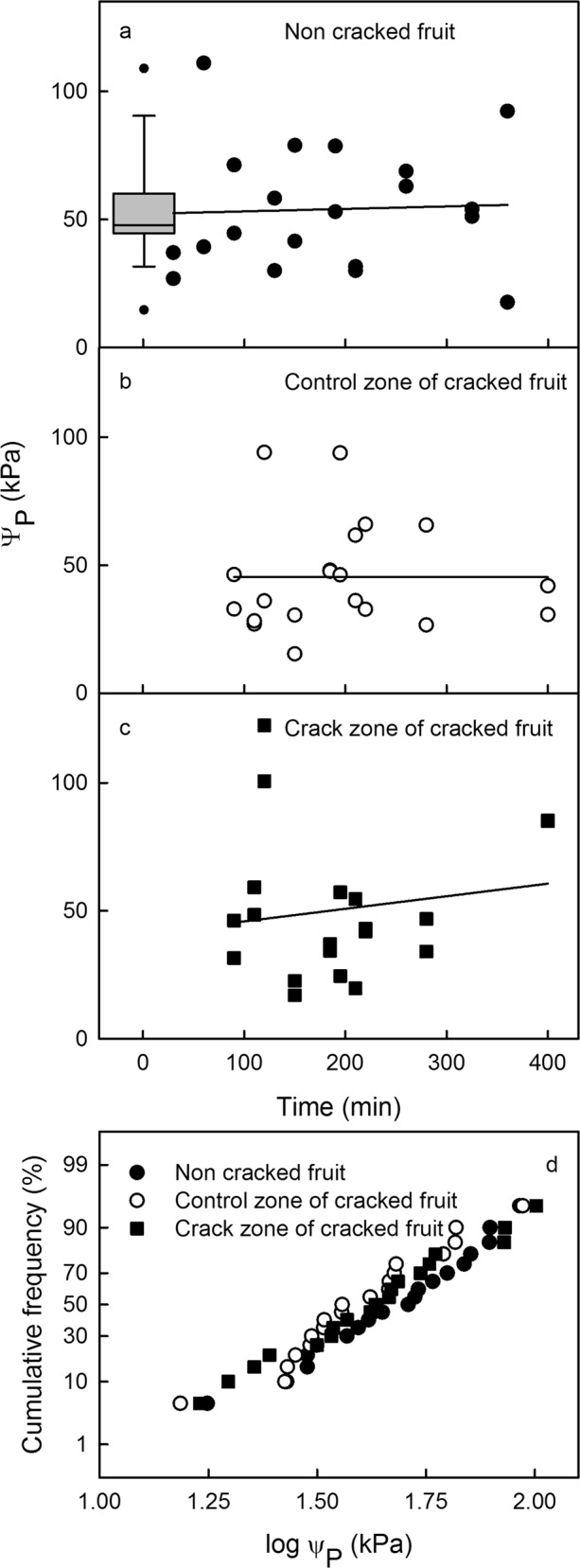
Cell turgor as affected by macroscopic cracking. **a**–**c** Time course of change in cell turgor as determined by the cell pressure probe (CPP). **a** Fruit that remains intact during incubation in water. The box plot represents data obtained in fruit before incubation. **b**, **c** Turgors of cells in the control zone, some distance from a macrocrack (**b**) and in the immediate vicinity of a macrocrack (**c**). **d** Cumulative frequency distributions of cell turgors of all cells shown in (**a**–**c**). Turgor follows a log normal distribution. There is no evidence of different populations of cells between the three conditions shown in (**a**–**c**). Relationships between cell turgor and incubation time were not significant. Data symbols in (**a**–**d**) represent individual cells

The turgors of the cells of the outer mesocarp were variable, covering a range of about one order of magnitude (Fig. 6). There were no significant differences in cell turgor before and after incubation of fruit in water. In cracked fruit, there were no significant differences in cell turgor between cells in the immediate vicinity of a macrocrack or those in a control zone some distance away from a macrocrack. The cumulative frequency distribution did not reveal any grouping of the different treatments indicating that all measured cell turgor pressures were from a single population of cells in the outer mesocarp.

## Discussion

Our results demonstrate that OCT, augmented by MRI, are useful tools for the nondestructive investigation of micro- and macrocracking in sweet cherry fruit. Moreover, these cracking phenomena are the result of a localized event, a microcrack, which develops into a macrocrack, whose subsequent propagation results in the destruction of the fruit.

### MRI and OCT—useful tools in cracking research

MRI is useful for the nondestructive monitoring of the movement of water, of D_2_O and of the tracer gadoteric acid in fleshy fruit^17–20^. When incubating fruit in D_2_O, the technique resolves the different regions of a sweet cherry fruit where D_2_O penetrates and accumulates. However, information on details of the vascular system requires different MRI settings or the use of gadoteric acid as a tracer^17^.

OCT proved to be a powerful tool for the nondestructive measurement and analysis of cell-level dimensional changes in whole fruit, submerged in water. This tool is particularly useful in fruit types, such as sweet cherry, where the skin is subject to elastic stresses and strains, such that the isolation of a skin sample is inevitably associated with an artifactual release of both stress and strain^21^. It is important to note that OCT analysis is restricted to a tissue depth of a maximum of about 500 µm in mature sweet cherry. This corresponds to the region of the fruit skin (about 100 µm; refs. ^22,23^) and the outer mesocarp. For studies on micro- and macrocracking this limitation is not a problem. First, we carried out incubation assays on detached, whole fruit that simulate rain events. Under these conditions, fruit water uptake was exclusively through the fruit skin—it did not include any uptake/loss component via the xylem and phloem vasculature of the fruit stalk. Second, cracking is a surface phenomenon: microcracking occurs generally in the cuticle, macrocracking extends microcracks into the epidermis and hypodermis and into the underlying mesocarp. The cuticle is the penetration barrier, epidermis and hypodermis form the structural backbone^24^. The resolution of the OCT was insufficient to resolve individual epidermal cells. These are very much smaller than those of the large thin-walled parenchyma of the outer mesocarp^22^. Unfortunately, the chances are low that a microcrack will be initiated and develop into a macrocrack at a highly localized site while it is under observation by OCT. Furthermore, for a meaningful OCT analysis, only single cracks must form (not multiple ones) in an area of known high cracking probability that also has a maximum radius of curvature. This requires use of a cracking-susceptible cultivar and careful optimization of experimental conditions. Nevertheless, the method is somewhat wasteful, as a large number of replicates are required and numerous and extensive 3D datasets must be collected, of which only a small portion will be of any value.

The fact that the experiments were carried out using different cultivars is an advantage rather than a limitation. First, sweet cherry is a rapidly maturing, highly perishable crop. Any one cultivar is available at the optimum stage of maturity for only a few days. Hence, a change of cultivars is mandatory when pursuing a complex objective with a set of experiments. In addition, experiments have to be repeated to ensure reproducibility and a sufficient number of replicates. Second, light microscopy and OCT are best performed on light colored genotypes fruit. These bear yellow fruit, for example of ‘Dönissens Gelbe’, or ‘Rainier’ fruit with a light blush. A dark colored cultivar (‘Sam’) was used for MRI. Third, there is no indication for cultivar-specific interactions for the processes studied and the change of cultivar ensures that conclusions are not limited to a single cultivar.

### The localized bursting of a mesocarp cell(s) triggers fruit macrocracking

Our data support the hypothesis that macrocracking results from a very localized increase in water uptake through a microcrack and the associated very localized bursting of an individual outer-mesocarp cell(s). As predicted by the zipper hypothesis, the failure of that single cell(s) concentrates the elastic stress in the skin onto its immediate neighbors. That initial cell failure also releases malate into the cell-wall-free space which weakens the walls of the adjacent cells. Together these outcomes cause the microcrack to “run”—i.e. to extend both tangentially along the skin and radially down into the mesocarp, to form a macrocrack. This conclusion is based on the following arguments.

First, localized water uptake occurred in the two polar regions of the fruit that, this and earlier studies^13^ show, suffer the highest frequency of microcracking. The frequent microcracking in these regions is the result of one (or several) of the following factors: (1) Skin stresses are concentrated due to the presence of the relatively rigid stylar scar (distal pole) and the pedicel attachment (proximal pole)^25^—the elastic moduli of cuticle and epidermis usually being lower than of periderm^26^. (2) The smaller radii of curvature of the polar surfaces relative to the equatorial ones^27^. (3) There is considerable strain in the skin and in the cuticle of mature sweet cherry fruit, the latter is due to a lack of cuticle deposition during the late phase of fruit development^21,28^. In addition, exposure of a strained cuticle to water aggravates microcracking probably as a result of a change of the cuticle’s mechanical properties upon hydration^9^. (4) The higher water permeability of the skin surface in this region^29^. In addition, after rainfall or heavy dew, a field-grown fruit suffers longer periods of wetness in its stylar scar region, where a pendant drop attaches and also in its pedicel cavity region, where a water “puddle” collects^9^. Whether or not localized expansion of the fruit skin causes microcracking in the stylar scar region of a sweet cherry, as has been postulated in grapes, is not known^30^. In our earlier studies we found stored elastic strain in the stylar scar region was lower than in the cheek region of the fruit^21,28^.

Second, while water uptake through the remaining surface of the fruit will likely have occurred, its rates remained below the detection limit of our MRI. Consistent with this is the large increase in the volumes of cells in the immediate vicinity of a microcrack and a developing macrocrack, compared with the absence of significant volume change in the control zone a short distance away. The fact that D_2_O uptake remained localized indicates the presence of significant internal resistance to water movement. This was also observed in earlier studies where a marked difference (1.1 MPa) in osmotic and water potentials occurred between skin and flesh^15^. The reason for the significant resistance to water diffusion is unclear. Several mechanisms may be visualized including a decreased mean free path of water due to swelling thus slowing its diffusion in the gelled apoplast. Alternatively, the overall path length is increased by the swelling. Or, lastly, the proportion of liquid water (diffusible) to bound water (nondiffusible) in the cell-wall-free space is sharply reduced by gelling. To our knowledge, the permeability of a swollen cell wall has not been quantified.

Third, the deepening of a macrocrack into the underlying cell layers occurs by separation of adjacent cells. This suggests failure along cell walls by separation of adjacent cells as predicted by the zipper hypothesis. This failure mode is consistent with findings in earlier studies where the predominant mode of fracture was along the middle lamella in both naturally cracked fruit and also in fruit-skin samples with swollen cell walls subjected to biaxial tensile testing^12^. Swelling of cell walls decreases their modulus of elasticity and decreases their stress at fracture, thereby facilitating the separation of adjacent cells.

Fourth, throughout the development of a macrocrack, there was no consistent change in cell turgor—either in submerged fruit that remained intact or in submerged fruit that cracked. Furthermore, cell turgor did not differ significantly between noncracked fruit and macroscopically cracked fruit regardless of whether cells in the control zone or in the crack zone were measured. Similarly, water uptake or transpiration had no effect on fruit turgor^7^. This behavior also is consistent with the zipper hypothesis. The lack of significant cell turgor relative to the very negative osmotic potential results from the low moduli of elasticity of the cell walls in mesocarp and skin. In addition, leakage of cell contents into the apoplast upon bursting of an individual cell eliminates the osmotic potential difference between apoplast and symplast in the immediate vicinity.

The above sequence of events accounts for rain-induced cracking where the fruit surface comes into contact with water. Occasionally, a small percentage of fruit also cracks under a rain shelter that protects the fruit surface from wetness^31^. In these cases, macroscopic cracking is not necessarily preceded by microcracking of the cuticle.

## Conclusion

These results provide direct evidence that macrocracking is the result of highly localized water uptake through cuticular microcracks, particularly in the stylar scar region of the sweet cherry fruit. The localized water uptake is linked to a localized failure (bursting) of a very few cells in the outer mesocarp. This initial, minor damage, in conjunction with an already stressed (taut) skin serves to focus the skin stresses onto the adjacent healthy cells. Meanwhile, the liberation of malic acid consequent of the initial cell failure weakens these cell walls. These processes together serve to unzip the tissue—a sort of domino effect. The situation is exacerbated by a continued (even increased) water uptake which causes the initiating microcrack to extend (lengthwise) round the fruit and to deepen (radially) into the underlying tissues. So develops the familiar fruit macrocrack. It is also important to note that cracking is neither preceded nor accompanied by any detectable change in fruit turgor. The events and processes identified here are likely to apply also to other cracking-susceptible fleshy fruit.

## Materials and methods

### Plant material

Mature sweet cherry (*Prunus avium* L., ‘Dönissens Gelbe’, ‘Rainier’ and ‘Sam’) were sampled from a greenhouse or under a rain shelter at the Horticultural research station at Ruthe (52.2 N, 9.8 E). For MRI measurements potted ‘Sam’ trees were cultivated under a rain shelter at the Forschungszentrum Jülich (50.9 N, 6.4 E). All trees were grafted on ‘Gisela 5’ rootstocks. All fruit were selected for uniformity based on skin color and freedom from visual defects.

### Cracking assays

The fruit of ‘Dönissens Gelbe’ was used for the cracking assays. The pedicel was cut back to a length of 5 mm. The fruit was incubated in deionized water at 22 °C within 24 h of sampling and regularly inspected for macroscopic cracks up to 24 h.

Formation of microcracks was quantified by incubating a fruit for 5 min in 0.1% acridine orange solution. Thereafter, the fruit was carefully blotted using tissue paper and then transferred to the stage of a microscope (MZ10; Leica Mikrosysteme, Germany) and viewed under incident fluorescent light using a GFPplus filter. Digital photographs were taken (DP73; Olympus Deutschland, Germany) and processed by image analysis (cellSens Dimension 1.7.1; Olympus).

### Optical coherence tomography measurement

The fruit of ‘Dönissens Gelbe’ was carefully selected for the absence of any visual defects in the stylar scar region. The pedicel cavity was sealed with silicone rubber (SE 9186; Dow Corning, MI, USA) to eliminate any water uptake and, hence, cracking in this region. Subsequently, the fruit was mounted in a small water bath such that the stylar scar region faced the OCT lens. Ten fruits were arranged in this position such that their stylar scar regions were in the same horizontal plane. The maximum time period between sampling from the field and the beginning of OCT scanning was 2 d during which the fruit was held at 2–4 °C.

The OCT system comprised a lens (LSM03-BB; Thorlabs, NJ, USA), a pair of galvanometer scanning mirrors (6210H; Cambridge Technology, MA, USA), a superluminescent diode having a central wavelength of 835 nm (BLMS‐mini‐351‐HP2‐SM‐I; Superlum Diodes, Ireland) and a long-range spectrometer (Cobra UDC; Wasatch Photonics, NC, USA). Image acquisition and processing was performed using the custom-made laboratory framework smartLab (Laser Zentrum Hannover, Germany). Settings for measurements were: 500 B-scans per fruit, a maximum depth of penetration into the fruit of ca. 500 µm, a field of view of 500 × 500 × 2048 voxels corresponding to 9370 × 9370 × 8400 µm^³^ in air, recording time for a full 3D image set <1 min per fruit. A total of 3 × 10 fruit were scanned.

The experiment was initiated by filling the bath with deionized, degassed water thereby submerging all the fruit. The stylar scar region of each fruit was scanned, one by one, for all ten fruits using OCT. Thereafter, the next scan cycle was initiated.

For data analysis, regions of interest (ROI) were chosen for each fruit that cracked in a location that enabled analysis. These ROIs were (1) the crack zone where a macrocrack developed from a microcrack during incubation and (2) a control zone that remained crack free. In both ROIs cells that had clearly detectable cell-wall boundaries in all planes were selected.

The images of the ROIs were processed by magnifying and filtering using the software package Fiji/ImageJ 1.51j (ref. ^32^). The resulting ROI had 1000 × 1000 × 500 voxels where 1 voxel corresponded to a volume of 9.37 × 9.37 × 3.09 µm^3^ (271.1 µm^3^) using the appropriate corrections for light refraction in water at the respective temperature (*n* = 1.3281, *λ* = 835 nm, *T* = 19 °C; ref. ^33^). For measurement of cell volumes, the cells were segmented starting from the center of a cell in 5−10 slices up to the cell wall in all directions using the software ITK-SNAP 3.6.0 (ref. ^34^). Missing data were interpolated and manual corrections were made where necessary. Using this procedure, four cells in the immediate vicinity of a developing macrocrack were selected and segmented at three stages of crack development: (i) at time *t* = 0 when the experiment was initiated, (ii) immediately (<30 min) before a crack became visible and (iii) at a maximum of 30 min after the crack had developed. Three cells in the control zone distant from the crack zone served as control.

### Magnetic resonance imaging

Mature ‘Sam’ fruit was sampled, the pedicel was cut close to the receptacle and the pedicel cavity was sealed using silicone rubber (SE 9186; Dow Corning). The silicone was allowed to cure for 100 min. The fruit was subsequently mounted in a custom-built holder and submerged in deuterium oxide (D_2_O; Sigma-Aldrich, Germany). Uptake and distribution of D_2_O (Sigma-Aldrich) were monitored beginning 25 min after submersion using MRI. The setup comprised a 4.7 T magnet (Magnex, UK) equipped with a Varian console (Varian, CA, USA) and a birdcage radio frequency coil with 100 mm inner diameter (Varian) (for details, see ref. ^35^). The custom-built sample holder comprised several trays for submerging fruit in D_2_O. The holder was positioned by an industrial pick-and-place robot (Mini-Liner 3.0 Alu; Geiger Handling, Germany) mounted on top of the magnet. This setup allowed repeated observations of the same fruit without removal of the sample holder. Hence, time courses of uptake and distribution of D_2_O could be established simultaneously on an individual fruit basis. The following settings were used: Spin-Echo Multi-Slice sequence, slice resolution = 0.336 × 0.336 mm^2^, field of view = 86 × 86 mm^2^, slice thickness = 0.7 mm, 150 slices, TR = 3500 ms, TE = 15 ms, spectral width = 50 kHz, two averages, acquisition time per cycle ~30 min.

MRI images were processed using image analysis software (MeVisLab 2.6.1.; MeVis, Germany). The individual time points of the series were normalized relative to the integral signal of the respective image, to correct for temporal variations of the signal due e.g. to temperature changes. The D_2_O diffusing into the fruit reduced the signal. The D_2_O distributions in virtual sections were thus calculated by subtracting the signal obtained on a section at each “feeding” time from the background signal of the same fruit for a section at time zero. The differential signal was expressed as heat maps with black/blue indicating no D_2_O accumulation and orange indicating high D_2_O accumulation. Negative differences are indicated in blue.

### Quantifying cell turgor using the cell pressure probe (CPP)

‘Rainier’ fruit was sampled and transferred to the laboratory. The pedicel was cut at the receptacle and the pedicel cavity, the pedicel−fruit junction and the stylar scar region were sealed with silicone rubber (SE 9186; Dow Corning). Turgor was quantified before and after incubating the fruit in deionized water. Measurements before incubation were made on the day of sampling. Fruit incubated in water for water uptake and crack induction was covered with moist tissue paper and held overnight at 2 °C. Incubation was initiated next morning. Turgor was determined in parenchyma cells of the outer mesocarp at depths below the surface of approximately 200–400 µm. Measurements in fruit with macrocracks were taken 2–4 mm away from the crack. For comparison, measurements were also made on the opposite side of the same fruit—i.e. at a maximum distance away from the macrocrack. Turgor was quantified using a CPP^36^. The instrument comprised a glass capillary filled with silicone oil that was connected to a pressure transducer (26PCGFA6D; Honeywell Sensing and Control, MN, USA). The glass capillary was carefully inserted into the fruit under a horizontal microscope using a micromanipulator. The peak pressure in the system was recorded as described previously^7,8^. Only those insertions were analyzed that fulfilled the following conditions: (1) The seal between capillary and cell must remain leak-free when subjecting cells to small, transient increases in pressure. (2) The pressure must return to the ambient value (i.e. that just before the insertion) when the capillary is withdrawn from the fruit. If these conditions were met, the peak pressure recorded in the oil was taken as an estimate of cell pressure (*ψ*_P_).

### Data analysis

Unless individual observations or boxplots are shown, data are shown as means ± standard errors. Where not visible, error bars are smaller than the data symbols. In boxplots the upper and lower ends of the box represent the 25 and 75 percentiles, and the line in the box the median. The bars give the 10 and 90 percentiles, the data symbols mark outliers. Data were analyzed by analysis of variance and regression analysis using the statistical software package SAS (version 9.1.3; SAS Institute, NC, USA).

## Supplementary information

Dataset 1

## Data Availability

The data that support the findings of this study are available from the corresponding author upon reasonable request.
